# Elastic Waves Excitation and Focusing by a Piezoelectric Transducer with Intermediate Layered Elastic Metamaterials with and without Periodic Arrays of Interfacial Voids

**DOI:** 10.3390/s23249747

**Published:** 2023-12-11

**Authors:** Mikhail V. Golub, Sergey I. Fomenko, Pavel E. Usov, Artem A. Eremin

**Affiliations:** Institute for Mathematics, Mechanics and Informatics, Kuban State University, 350040 Krasnodar, Russia; sfom@yandex.ru (S.I.F.); Lyova-pavel.usov@yandex.ru (P.E.U.); eremin_a_87@mail.ru (A.A.E.)

**Keywords:** elastic metamaterial, piezoelectric transducer, elastic waves, voids, periodic array, mode conversion, wave energy, laminate

## Abstract

Optimization of the structure of piezoelectric transducers such as the proper design of matching layers can increase maximum wave energy transmission to the host structure and transducer sensitivity. A novel configuration of an ultrasonic transducer, where elastic metamaterial insertion is introduced to provide bulk wave mode conversion and to increase wave energy transfer into a substrate, is proposed. Configurations of layered elastic metamaterials with crack-like voids are examined theoretically since they can provide wide band gaps and strong wave localization and trapping. The analysis shows that the proposed metamaterial-based matching layers can sufficiently change wave energy transmission from a piezoelectric active element for various frequency ranges (relatively low frequencies as well as higher ones). The proposed configuration can also be useful for advanced sensing with higher sensitivity in certain frequency ranges or for demultiplexing different kinds of elastic waves.

## 1. Introduction

For the routine nondestructive inspection of metallic and composite structures, ultrasonic methods relying on elastic waves as a physical basis are widely adopted [1,2,3,4,5]. A common way for the excitation of wave motion in the examined construction is to use piezoelectric transducers of different types. Typical commercially available ultrasonic probes could provide only narrowband excitation and are not designed for the simultaneously efficient generation of all necessary kinds of elastic waves [6,7]. At the same time, increasing complexity of the materials from which modern structures are made, dictates the necessity of the advancements in the transducer design which could address these issues.

Progress in additive manufacturing and computations has stimulated the design and engineering of various advanced materials that have led to the rapid development of many novel structures and systems with previously unforeseen characteristics or superior performance [8,9]. Metamaterials, which can be referred to as rationally designed composites with properties exceeding those of their constituents [9,10], are among them. In particular, elastic metamaterials (EMMs) are composite elastic materials with artificial microstructures made to exhibit unusual mechanical wave characteristics such as waveguiding, wave focusing and lensing, energy conversion etc. An original metamaterial classification for civil engineering applications was recently presented based on the types of waves targeted for mitigation Contreras et al. [11]. On the other hand, ultrasonic waves have been widely applied for non-destructive evaluation (NDE) and structural health monitoring (SHM) of engineering structures because of their considerable sensitivity to possible faults such as cracks, pitting corrosion, voids and delaminations [12,13]. Since EMMs can be used to manipulate elastic wave propagation, EMM employment as elements of transducers and sensors seems to have a significant potential. The latter is beyond the usual scope of NDT and SHM, whereas EMMs can be designed and optimized to provide the required properties, e.g., wider band-gaps, specific resonances or negative refractive index [14].

Common ultrasonic transmitting and sensing transducers can consist of actuating piezoelectric elements and wedges introduced to target elastic wave energy in the preferable direction. However, there is still a lack of proper design to bridge the energy generated by a piezoelectric actuator and the target elastic medium over the wide operating spectrum [15]. The design of such devices needs to be optimized in order to maximize the energy transfer efficiency [16]. Thus, Li et al. [16] developed an anisotropic cone-structured EMM layer for matching piezoelectric actuator and substrate for improvement of broadband ultrasound transducers. Mohammadgholiha et al. [17] presented a frequency steerable acoustic transducer with spiral electrodes based on the frequency-dependent spatial filtering effect to generate directional guided waves in a host structure.

Since mode conversion can drastically change elastic wave energy transfer, many recent studies were focused on the conversion efficiency increase and the integration of converters as parts of devices and structures [18,19,20]. Methods based on Snell’s law showed full mode conversion for double-negative and triple-negative transmitted media [21], but they are strongly restricted by the conditions needed to be satisfied [22]. Perfect transmodal Fabry–Perot interference theory and impedance matching theory were later proposed to implement high mode-conversion induced by stiffness anisotropy [23,24]. For longitudinal and transverse waves, Chai et al. [25] proposed asymmetric mode-converting EMM for full conversion of one mode to another in one direction and severely restricted the wave transmission in the opposite direction. Chai et al. [26] have demonstrated numerically full mode-converting transmission between longitudinal and bending waves in thin plates and Euler–Bernoulli beams in narrow low frequency ranges. Lee et al. [27] presented a theory for full wave energy transmission through solid–solid interfaces and proposed a non-resonant anisotropic single-phase EMM, which realizes the theory. Anisotropic mass density can be based on non-resonance mechanism, which are mostly used in fluid–solid metamaterials, whereas local resonances are usually employed in purely elastic metamaterials [22]. Piao et al. [19] demonstrated that EMM in the form of two periodic arrays of slits can completely convert wave longitudinal waves into transverse waves in the forward direction and forbid longitudinal wave transmission in the inverse direction. Therefore, they showed that such EMM can improve mode-converting transmission efficiency.

Nevertheless, the EMM mentioned above and many others can support wave phenomena (conversion, focusing etc.) in relatively narrow frequency ranges, usually not more than 10–20 kilohertz [19] or even less [25]). The optimization of the structure of ultrasonic transducers (e.g., proper design of matching layers between piezoelectric active element and substrate) could provide maximum wave energy transmission and increase transducer sensitivity [28]. We propose and numerically examine here a novel configuration of a wedge transducer, where EMM insertion is introduced to provide mode conversion and to increase the wave energy transfer into a substrate. Configurations of layered EMMs with crack-like voids are compared here with a traditional configuration with a single piezoelectric actuator since this kind of inhomogeneity can provide strong wave localization and wave energy trapping [29,30] as well as wide band-gaps [31], which might be employed to enhance the characteristics of ultrasonic transducers.

## 2. Formulation of the Problem

### 2.1. Aim and Design

**Figure 1:**
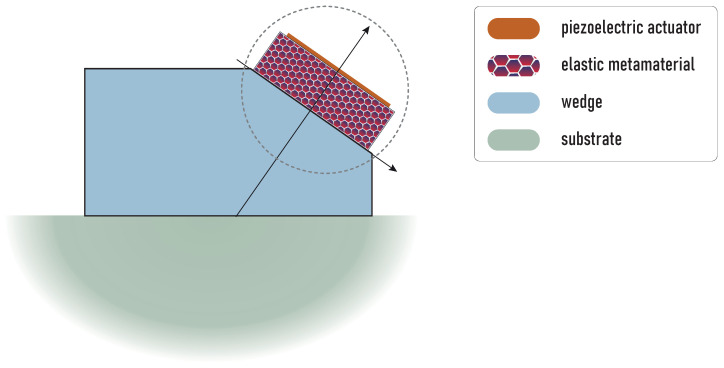
An example of possible design of the proposed configuration of the ultrasonic transducer with the piezoelectric actuator and EMM intermediate.

The aim of this study is to consider the employment of EMM for providing advanced characteristics of wedge transducers. A schematic design of the considered transducers with EMM is depicted in Figure 1. Here, EMM intermediate is inserted between the piezoelectric active element and the wedge to change the impedance and to manipulate the excitation of the required kind of elastic waves in the media.

For wave propagation analysis, efficient and accurate mathematical models and numerical simulation tools should be developed for fast parametric studies at the development stage or the implementation in real electromechanical devices and systems. Let us consider wave excitation in the wedge by the rectangular piezoelectric actuator with an intermediate laminated rectangular EMM block as schematically shown by a dashed circle in Figure 1. In the present study, we employ the boundary integral equation method [29,32,33], the semi-analytical hybrid approach [34,35] and the finite element method to solve the boundary-value problem described in the following subsection.

### 2.2. Mathematical Statement of the Problem

**Figure 2:**
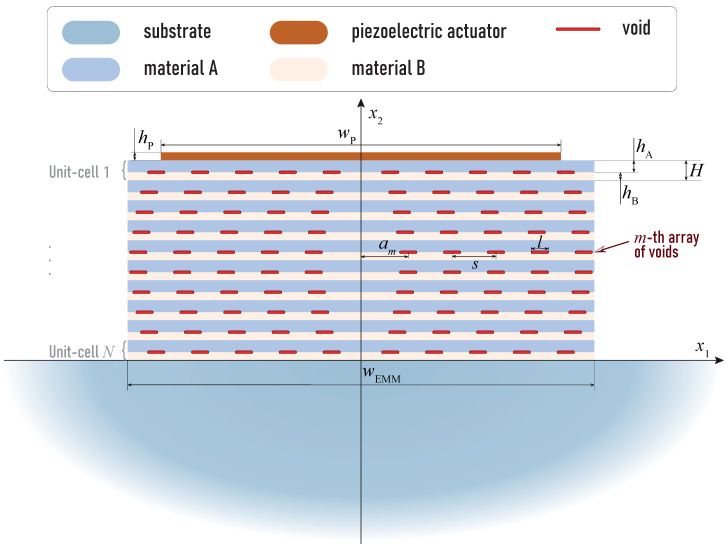
Geometry of the structure with a piezoelectric actuator and EMM with periodic arrays of interfacial voids.

The elastic isotropic half-space with a surface-mounted layered elastic block is considered. The rectangular block of width $w_{\mathrm{EMM}}$ consists of *N* unit-cells composed of two elastic layers (A and B) with thicknesses $h_{A}$ and $h_{B}$ ($H=h_{A}+h_{B}$) and may include a system of crack-like voids of widths *l* with spacing *s* at the layer interfaces (Figure 2). Elastic waves are excited by the piezoelectric actuator of width $w_{P}$ and height $h_{P}$ situated at the top surface of the EMM intermediate block. Let us assume that EMM is symmetric with respect to the $Ox_{2}$ axis, so one can define arrays of voids of thickness $h_{0}$ as follows: $\Omega_{m,j}^{\pm }=\left\{\right|x_{1}\mp a_{m}\mp js|\leq l/2,|x_{2}-h_{A}-\left(j-1\right)H\left|\leq h_{0}\right\}$.

The governing equations in an isotropic elastic media have the following representation in terms of displacement vector u:

(1) $$\left(\lambda +\mu \right)\nabla \left(\nabla \cdot u\right)+\mu \left(\nabla \cdot \nabla \right)u-\rho \frac{\partial^{2}u}{\partial t^{2}}=0.$$

The traction vector $\tau_{n}$ at the plane with the normal n can be expressed in terms of the displacement vector u and Lame constants μ and λ as follows:

$$\tau_{n}=\lambda n\nabla \cdot \nabla u+2\mu \frac{\partial u}{\partial n}+\mu \left(n\times \nabla \times u\right)$$

Elastic waves are excited in the considered assembly by the input electric voltage $V_{0}p\left(t\right)$ applied at the electroded upper surface of the piezoelectric actuator

$$\varphi \left(x_{1},H\cdot N,t\right)=0,\;\varphi \left(x_{1},H\cdot N+h_{P},t\right)=V_{0}\cdot p\left(t\right),$$

where φ is the electric potential and p(t) is the finite function of time. Wave motion in the piezoelectric material is governed by the following equations:

(2) $$\sigma_{ij,j}-\hat{\rho }\frac{\partial^{2}u_{i}}{\partial t^{2}}=0,\;D_{i,i}=0.$$

Here,

$$\sigma_{ij}=C_{ijkl}u_{k,l}+e_{kij}\varphi ,k,$$

$$D_{i}=e_{ikl}u_{k,l}-\varepsilon_{ik}\varphi_{,k},$$

$C_{ijkl}$ are the elastic constants, $e_{kij}$ and $\varepsilon_{ij}$ are the piezoelectric and dielectric constants, respectively, whereas $\hat{\rho }$ is the mass density of the material.

Displacement u and traction $\tau_{2}$ vectors are continuous at all of the interfaces $x_{2}=z_{n}$ between layers (including the piezoelectric actuator and the elastic half-space), as in the following:

$$\left[u\right]=0,\left[\tau_{2}\right]=0\;\;\mathrm{at}\;x_{2}=z_{n}.$$

The tension $\tau_{n}$ is zero at free surfaces of the block and the half-space, as well as it is zero at faces of the crack-like voids. Finally, the principle of limiting absorption [36] as the radiation conditions is assumed at infinity $x_{2}\to -\infty$ in the half-space.

Due to linearity of Equations (1) and (2), the integral transform with respect to time *t* in the form

$$u\left(x,\omega \right)=\int_{0}^{\infty }u\left(x,t\right)e^{i\omega t}dt.$$

can be used to exclude time derivatives and to consider separately steady-state harmonic motion with the angular frequency ω=2πf related with frequency *f*. It should be noted that the inverse Laplace transform

$$u\left(x,t\right)=\frac{1}{\pi }Re\left[\int_{0}^{\infty }u\left(x,\omega \right)P\left(\omega \right)e^{-i\omega t}d\omega \right].$$

is applied to the time-harmonic solution u(x,ω). Here, P(ω) is the Laplace transform of the input voltage signal p(t).

In the case of harmonic motion with the angular frequency ω, Equations of motion (1) and (2) have the following form for elastic isotropic and piezoelectric media [37,38]:

$$\left(\lambda +\mu \right)\nabla \left(\nabla \cdot u\right)+\mu \left(\nabla \cdot \nabla \right)u+\rho \omega^{2}u=0,$$

$$\sigma_{ij,j}+\rho \omega^{2}u_{i}=0,\;D_{i,i}=0.$$

Material parameters of the elastic isotropic sub-layers and piezoelectric actuator made of PIC155 (modified lead zirconate titanate) from PI Ceramic [39] used in the numerics below are given in Table 1.

## 3. Transducer with EMM Intermediate without Voids

### 3.1. Plane Wave Propagation through a Periodic Layered Medium

First, let us consider the interaction of the piezoelectric active element with the EMM intermediate without voids. Though the wave propagation in the elastic block of finite width has specific features due to reflections from its side surfaces, band-gaps and pass-bands in the periodically laminated block can be determined via the consideration of unbounded periodic media. Namely, plane wave propagation through an unbounded multi-layered structure, i.e., $w_{\mathrm{EMM}}=\infty$, can be considered here.

To predict the allocation of pass-bands and forbidden zones, one can study the plane wave propagation through a typical one-dimensional phononic crystal (PnC) or layered EMM composed of elastic layers

$$L_{A,n}=\left\{\left(x_{1},x_{2}\right)\;|\;x_{1}\in R,\;H\left(N-n\right)+h_{B}<x_{2}⩽H\left(N-n+1\right)\right\}$$

and

$$L_{B,n}=\left\{\left(x_{1},x_{2}\right)\;|\;x_{1}\in R,\;H\left(N-n\right)<x_{2}⩽H\left(N-n\right)+h_{B}\right\},$$

which are made of materials denoted *A* and *B*, respectively. The layered periodic structure of *N* unit-cells is assumed to be situated between two elastic half-planes, whose properties are the same as those of the half-space. The steady-state harmonic motion in the structure is excited by a normally incident plane wave propagating from the top half-plane.

#### 3.1.1. Transfer Matrix Method

The transfer matrix method is more suitable for analysis of plane wave propagation in the considered structure since it gives simple expressions for elastic wave motion in homogeneous elastic sub-layers $L_{k}=\left\{\left(x_{1},x_{2}\right)\;|\;x_{1}\in R,\;z_{k}⩽x_{2}⩽z_{k-1}\right\}$ in terms of the generalized state vector $v\left(x_{2}\right)=\left\{u,\tau_{2}\right\}$ as follows:

(3) $$v\left(z_{k}\right)=T_{k}v\left(z_{k-1}\right),$$

where $T_{k}$ is the transfer matrix-function of the layer $L_{k}$. The transfer matrix can be expressed explicitly in terms of the elastic constants and the angular frequency ω; see, for instance [40,41] for more details. Using the definition (3) of the transfer matrix and the continuity of the displacement vector $v(x_{2})$ at the interfaces, the transfer matrices of the unit-cell $T_{c}$ and a matrix $T_{PnC}$ of the whole PnC composed of *N* unit-cells are written as matrix product

$$T_{c}=T_{B}T_{A},$$

$$T_{PnC}={\left(T_{c}\right)}^{N}=G^{-1}\Lambda^{N}G,$$

where $T_{A}$ and $T_{B}$ are the transfer matrices of the layers $L_{A}$ and $L_{B}$, respectively, G is a change-of-basis matrix to the Jordan diagonal form

$$\Lambda =\exp(i\;H\;\mathrm{diag}\left\{\zeta_{1},\zeta_{2},-\zeta_{1},-\zeta_{2}\right\})$$

of the unit-cell matrix $T_{c}$:

$$|T_{c}-\exp\left(i\zeta_{k}\;H\right)E|=0.$$

Here, E is the identity matrix 4×4, wavenumbers $\zeta_{k}$ are chosen in accordance to the following rule: Reζm⩾0,Imζm⩾0,ζm+2=−ζm and m=1,2.

Then the generalized state vector in the half-planes has the following view

$$v\left(x_{2}\right)=\left\{\begin{array}{cc} M^{+}r+v_{inc}, & \;x_{2}⩾HN,\\ M^{-}t, & \;x_{2}⩽0, \end{array}\right.$$

where $M^{\pm }$ are known matrices [41] of the eigensolution for P- and SV- waves in the corresponding half-planes, $v_{inc}$ is the incident field, t and r are amplitude coefficient vectors of waves transmitted and reflected by the laminated periodic structure that can be defined from the equation

$$v\left(0\right)=T_{PnC}v\left(HN\right).$$

The amplitudes t of the transmitted waves can be expressed in terms of eigenvalues of the transfer matrix as follows

(4) $$t=b_{1}e^{i\zeta_{1}\;H\;N}+b_{2}e^{i\zeta_{2}\;H\;N},$$

where components of vectors $b_{k}$ are bounded at N→∞, see [41,42] for more details.

#### 3.1.2. Energy Transmission Coefficient and Localization Factor

Relation (4) allows us to analyze the structure of band-gaps. Thus, if one of the wavenumbers $\zeta_{1}$ or $\zeta_{2}$ is not purely real (attenuation is observed if Imζk>0) in a certain frequency domain, the forbidden zone for the corresponding wave is observed (so-called longitudinal or transverse wave band-gap). If both wavenumbers $\zeta_{1}$ or $\zeta_{2}$ are complex numbers for a given frequency ω, this frequency corresponds to a band-gap.

For convenience and clarity, wave energy characteristics are further employed. The Umov-Pointing vector e is the time-averaged power density vector, whose components are expressed in terms of the dot product of displacement and traction vectors as follows: [43,44,45]

ei=−ω2Im[u·τi],i=1,2.

The energy transmission and reflection coefficients $\kappa^{\pm }=e_{2}^{\pm }/e_{2}^{0}$ are written in terms of the energy flux $e_{3}^{0}$ of the incident wave at the top half-plane, the energy flux $e_{2}^{-}$ of the reflected waves and the energy flux $e_{3}^{+}$ is the elastic wave energy transferred through the periodic structure. The energy conservation law is held as

$$\kappa^{+}+\kappa^{-}=1.$$

For the wave energy transmission coefficient, the following estimation is valid

κ+=Omaxn=1,2bn2e−2ImζnHN.

Moreover, the localization factor showing the exponential decaying in the band-gaps for waves propagating through the EMM can be introduced

$$\gamma =-\lim_{N\to \infty }\frac{\ln\kappa^{+}}{2H\;N}.$$

The localization factor γ depends on the type of incident wave. Therefore, the linear independent generalized state vectors $v_{inc,P}$ and $v_{inc,SV}$ for incident P-waves or SV-waves produce given localization factors γP=Imζ1 and γS=Imζ2, respectively, for longitudinal and transverse waves. Frequency bands with nonzero localization factor $\gamma_{P}$ are called longitudinal band-gaps, while frequencies where $\gamma_{S}>0$ belong to transverse band-gaps. The ranges, where the both of $\gamma_{P}$ and $\gamma_{S}$ are nonzero, correspond to band-gaps.

**Figure 3:**
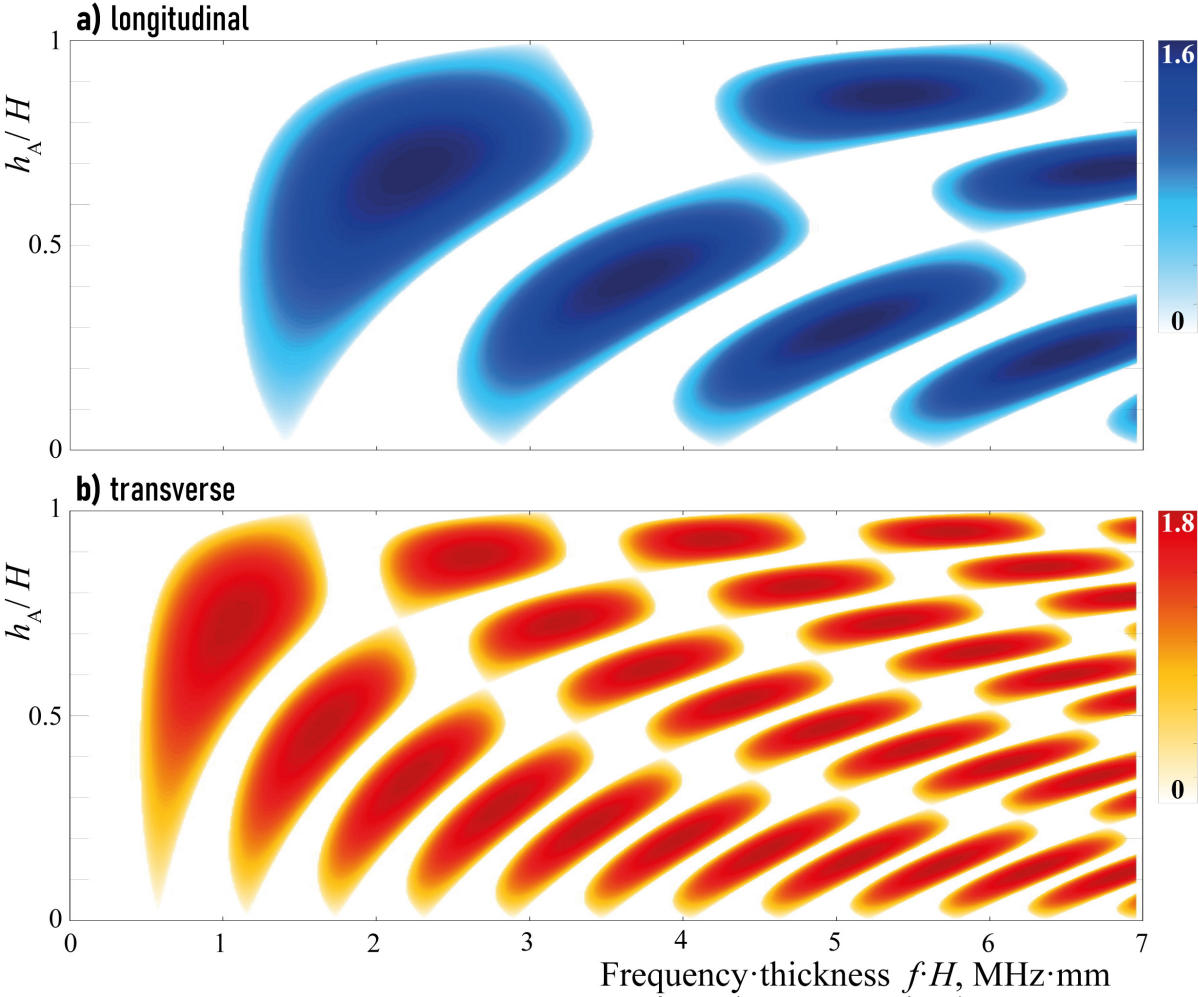
Localization factors $\gamma_{P}\left(f,h_{A}/H\right)$ (**a**) and $\gamma_{S}\left(f,h_{A}/H\right)$ (**b**).

**Figure 4:**
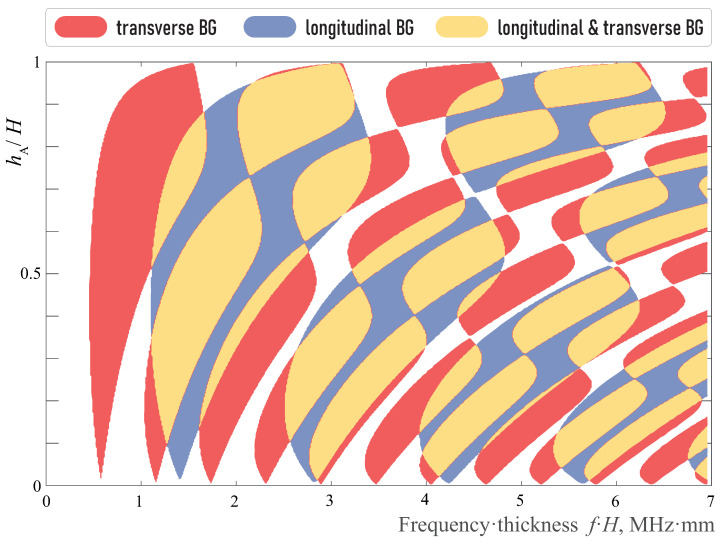
Longitudinal and transverse band-gaps dependence on $h_{A}/H$.

The influence of aluminium layer width $h_{A}$ in the unit-cell of total width *H* in the EMM on the localization factors $\gamma_{P}$ and $\gamma_{S}$ are shown in Figure 3. One can see that they intersect, i.e., band-gaps should also be distinguished. Figure 4 shows only band-gap locations for the three considered kinds with respect to the width $h_{A}/H$. With aluminum layer thickness increasing $h_{A}$, the band-gaps shift to higher frequencies and become wider.

**Figure 5:**
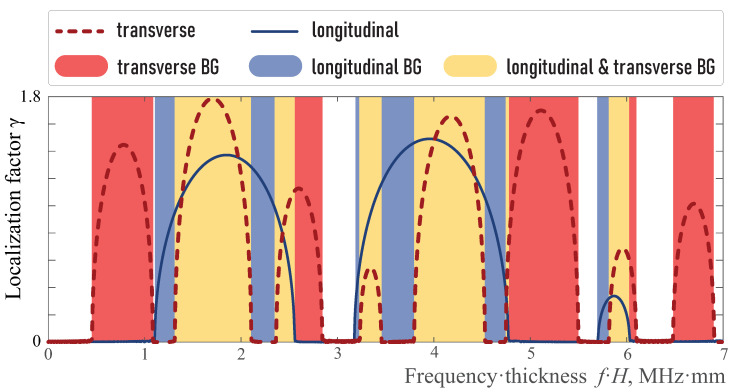
Localization factor γ(f) and band-gaps; $h_{A}=h_{B}$.

Without loss of generality, the case of a unit-cell with equal thickness of aluminium and epoxy sub-layers ($h_{A}=h_{B}$) is considered. Indeed, the change in the ratio $h_{A}/h_{B}$ does not sufficiently influence wave phenomena, but changes the frequency ranges and values of the corresponding localization factors. Thus, localization factors $\gamma_{P}$ and $\gamma_{S}$ for $h_{A}=h_{B}$ are presented in Figure 5.

### 3.2. Wave Propagation Excited by Piezoelectric Transducer through a Half-Plane with EMM Layers

#### 3.2.1. Semi-Analytical Hybrid Approach

The next model describes more accurately the stated boundary-value problem for a piezoelectric active element mounted on the surface of the EMM block. In this model, the block made of *N* unit-cells has infinite width ($w_{\mathrm{EMM}}=\infty$), while the transducer is of finite size. To solve this boundary-value problem of wave excitation and propagation for harmonic motion, the semi-analytical hybrid approach has been applied. Wave motion of a piezoelectric transducer with given voltage on surface electrodes is simulated using the spectral finite element method. Wave-field in the layered waveguide with regular boundaries is obtained in the integral form using the Fourier transform of the Green’s matrix of the layered structure and a load generated by the transducer [34,35]. The employed numerical algorithm for the evaluation of the Green’s matrix of layered elastic structures including periodic ones can be found in [33,46].

Displacement vector in the isotropic elastic half-plane has the following integral representation

$$u\left(x\right)=\frac{1}{2\pi }\sum_{n=1}^{2}\int_{\Gamma }K_{n}\left(\alpha ,x_{2}\right)Q\left(\alpha \right)e^{\sigma_{n}x_{2}-i\;\alpha x_{1}}d\alpha$$

where $K_{1}$ and $K_{2}$ are the parts of Green’s matrix responsible for body P- and SV-waves, respectively, Q is the Fourier transform of the surface load vector, $\sigma_{n}=\sqrt{\alpha^{2}-\varkappa_{n}^{2}}$ as well as

$$\varkappa_{1}=\omega /\sqrt{\lambda +2\mu }$$

and

$$\varkappa_{2}=\omega /\sqrt{\mu }$$

are wavenumbers of the P- and S-waves, respectively.

#### 3.2.2. Wave Energy Flux in Far-Field Zone

Applying the method of the stationary phase [47], the following asymptotics can be derived for the far-field displacement amplitudes $u_{P}=u_{1}$ and $u_{SV}=u_{2}$ [48]:

(5) $$u_{n}=C\cdot K_{n}\left(-\varkappa_{n}\cos\phi ,0\right)Q\left(-\varkappa_{n}\cos\phi \right)\frac{\exp\left(i\varkappa_{n}\;r\right)\sqrt{\varkappa_{n}}\sin\phi }{\sqrt{2\pi i\;r}},\;\varkappa_{n}\;r\gg 1.$$

Here, the matrix $C={\left[n_{r};n_{\phi }\right]}^{T}$ is composed of the normal vector $n_{r}=\left\{\cos\phi ;\sin\phi \right\}$ and the tangential vector $n_{\phi }=\left\{\sin\phi ,-\cos\phi \right\}$ in the polar coordinates $(x_{1}=r\cos\phi$, $x_{2}=r\sin\phi )$.

The energy fluxes of body waves are independently separated in the isotropic half-plane, similar to the body wave displacements. So, the energy flux through the circle sector between angles $\left(-\phi_{0},\phi_{0}\right)$ is given as

$$E_{V}\left(\phi_{0}\right)=E_{P}\left(\phi_{0}\right)+E_{SV}\left(\phi_{0}\right),$$

where

$$E_{\gamma }\left(\phi_{0}\right)=\int_{-\phi_{0}}^{\phi_{0}}e_{\gamma }\left(r,\phi \right)r\;d\phi ,\;\gamma \in \left\{P,SV\right\},$$

Here, $e_{P}$ and $e_{SV}$ are the projections of the Umov-Pointing vector e onto the normal to the circle of radius *r* with the center in the origin for longitudinal (P-waves) and transverse (S-waves) waves, respectively. Therefore, asymptotics for energy flux densities $e_{P}$ and $e_{S}$ can be expressed as

$$e_{P}=\frac{\omega }{2}\varkappa_{P}\left(\lambda +2\mu \right){\left|u_{P}\right|}^{2},$$

$$e_{SV}=\frac{\omega }{2}\varkappa_{SV}\mu \;{\left|u_{S}\right|}^{2}$$

at r→∞ employing asymptotic (5).

### 3.3. Analysis of Elastic Wave Energy Flux

**Figure 6:**
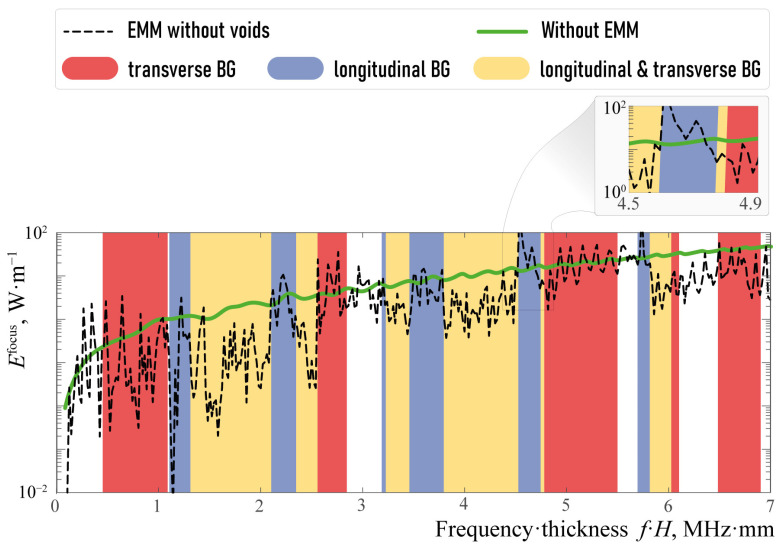
Elastic wave energy $E^{\mathrm{focus}}\left(f\right)$ generated by the piezoelectric transducer in the substrate $V_{0}$ and transferred in vertical direction for $h_{A}=h_{B}=2$ mm.

In the simulations presented below, the width and height of the piezoelectric actuating element have been chosen as $w_{P}=30$ mm and $h_{P}$ = 0.25 mm, respectively. Figure 6 demonstrates the frequency dependence of the elastic wave energy transferred from the piezoelectric transducer into the vertical direction

$$E^{\mathrm{focus}}=\int_{l^{\mathrm{focus}}}e_{2}\left(x_{1},x_{2}\right)dl.$$

The latter is calculated as the integral along the horizontal line

$$l^{\mathrm{focus}}=\left\{x_{1}\leq w_{P},x_{2}=-b_{P}\right\},$$

so $E^{\mathrm{focus}}$ is the total amount of the wave energy passed though $l^{\mathrm{focus}}$. Since $E^{\mathrm{focus}}$ is directly related to the acoustic impedance, these values might serve as an equivalent and more general value revealing intensive energy transfer from the transducer to the substrate for the investigated frequency ranges.

**Figure 7:**
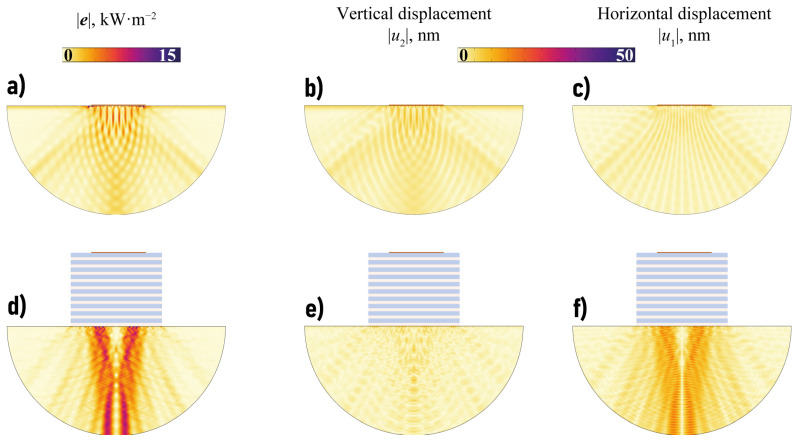
The power density vector |e(x)|, the horizontal and vertical components $|u_{k}\left(x\right)|$ of the displacement vector in the substrate in the case of the single piezoelectric actuator without EMM intermediate (**a**–**c**) and with EMM without voids (**d**–**f**) at ω=1144 kHz.

One can see in Figure 6 that the amount of wave energy transferred in a vertical direction with the EMM is larger compared to the case of the single piezoelectric actuator, mostly in some transverse or longitudinal band-gaps. For example, such a longitudinal band-gap can be clearly seen for fH∈[4.52,4.75] and transverse band-gap takes place if fH∈[4.78,5.49] MHz mm. Figure 7 shows the power density vector |e(x)|, the horizontal and vertical components $|u_{k}\left(x\right)|$ of the displacement vector in the case of the single piezoactuator and the setup with the EMM without voids at ω=1144 kHz (ωH=4.57 MHz mm). Figure 7 illustrates the fact that the horizontal component of the displacement vector and energy flux into the structure become much larger if the EMM is situated between the piezoelectric actuator and the substrate.

**Figure 8:**
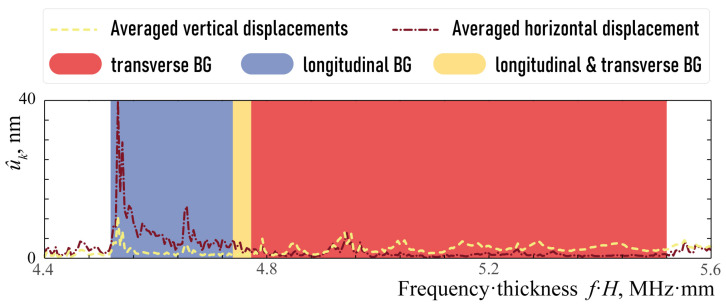
Averaged horizontal (dash-dotted line) and vertical (dashed line) displacements ${\hat{u}}_{k}$ corresponding to the wave energy transfer in the vertical direction.

The same conclusion regarding much higher horizontal displacements can be drawn considering averaged horizontal (dash-dotted line) and vertical (dashed line) displacements ${\hat{u}}_{k}$, see Figure 8. These integral characteristics describe the amplitudes of the wave-fields corresponding to the wave energy transfer in the vertical direction. They are computed in the area

$$S^{\mathrm{focus}}=\{x_{1}\leq w_{P},|x_{2}+b_{P}\left|\leq \varepsilon_{b}\right\}$$

situated in the vicinity of $l^{\mathrm{focus}}$, i.e.,

$${\hat{u}}_{k}=\int_{S^{\mathrm{focus}}}|u_{k}\left(x)\right|dS.$$

**Figure 9:**
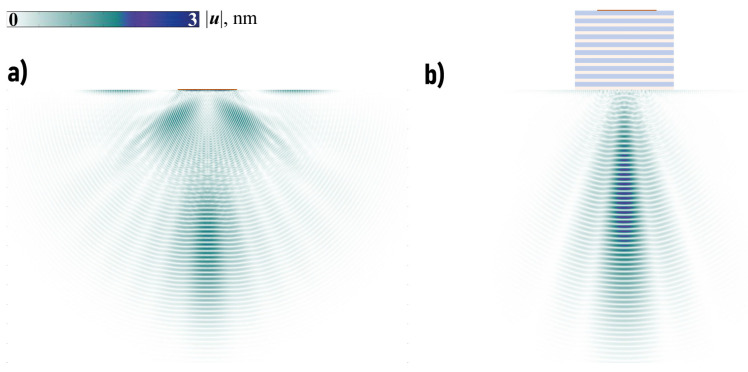
The distribution of amplitudes of the displacement vector u(x,t) for Hann-windowed transient signal at $f_{0}=1138$ kHz without EMM at t=0.55 ms (**a**) and with EMM without voids t=0.6 ms (**b**).

The snapshots of the transient solution corresponding to the input signal with a spectrum localized in the frequency range where focusing is observed for the time-harmonic problem are demonstrated in Figure 9. Namely, the amplitudes of the displacement vector |u| for $N_{c}$-cycled Hann-windowed signal with the central frequency $f_{0}=1138$ kHz

(6) $$p\left(t\right)=\frac{1}{2}\cos\left(2\pi f_{0}t\right)\left(1-\cos\left(\frac{2\pi f_{0}t}{N_{c}}\right)\right),\;0<t<\frac{N_{c}}{f_{0}}$$

are shown for both cases at different times to have approximately the same arrival time at the observation point ($N_{c}=20$). The introduction of the EMM has provided better focusing of the wave energy into the structure and larger amplitudes if the spectrum of the input signal p(t) is chosen according to the analysis of the harmonic problem.

## 4. Transducer with EMM Intermediate with Arrays of Crack-like Voids

In the case of EMM with arrays of voids, the analysis similar to the one provided in the previous section can be performed. Based on the previous investigations [31], three different configurations have been considered: hexagonal and rectangular lattices as well as an oblique lattice with a rhombic channel without voids. For the rectangular lattice, $a_{m}=s/2$, whereas $s=2H/\sqrt{3}$, $a_{2m}=0$ and $a_{2m-1}=s/2$ for the hexagonal lattice. In the case of the centers of the cracks lying on a rhombus with angles ψ and π−ψ, for the centers of the first M/2 cracks ($m=\bar{1,M/2}$) in *m*-th layer, one can state

$$a_{m}=a_{0}/2+\left(h_{A}\left(m-1\right)+mh_{B}\right)\cot\psi -l/2,$$

while the centers of the last voids ($m=\bar{M/2+1,M}$) have the coordinates

$$a_{m}=a_{0}/2-\left(h_{A}\left(M+1-m\right)+\left(M-m\right)h_{B}\right)\cot\psi -l/2.$$

**Figure 10:**
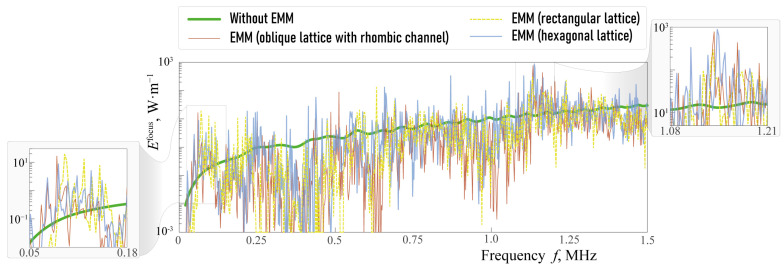
Elastic wave energy $E^{\mathrm{focus}}\left(f\right)$ generated by the piezoelectric transducer in the substrate and transferred in vertical direction for $h_{A}=h_{B}=2$ mm.

**Figure 11:**
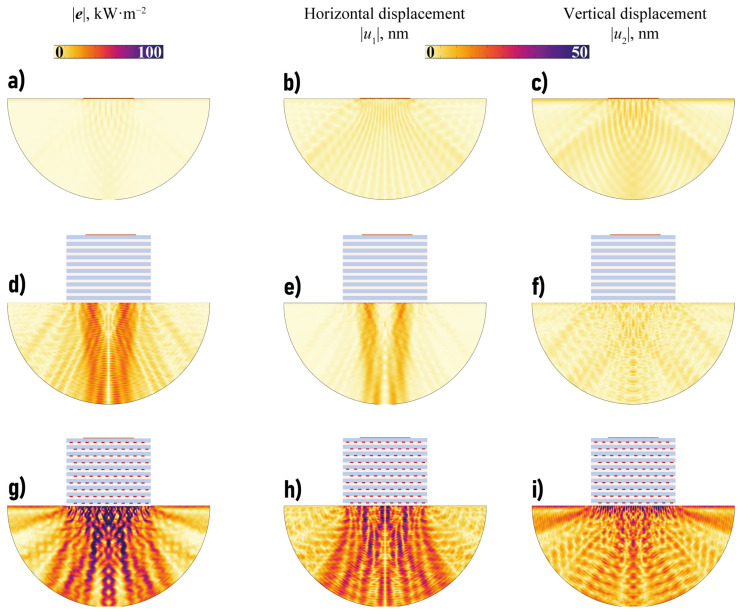
The power density vector |e(x)|, the horizontal and vertical components $|u_{k}\left(x\right)|$ of the displacement vector in the substrate in the case of the actuator without EMM intermediate (**a**–**c**), with EMM intermediate without voids (**d**–**f**) and with voids in hexagonal lattice l=1.75 mm at f=1138 kHz (**g**–**i**).

**Figure 12:**
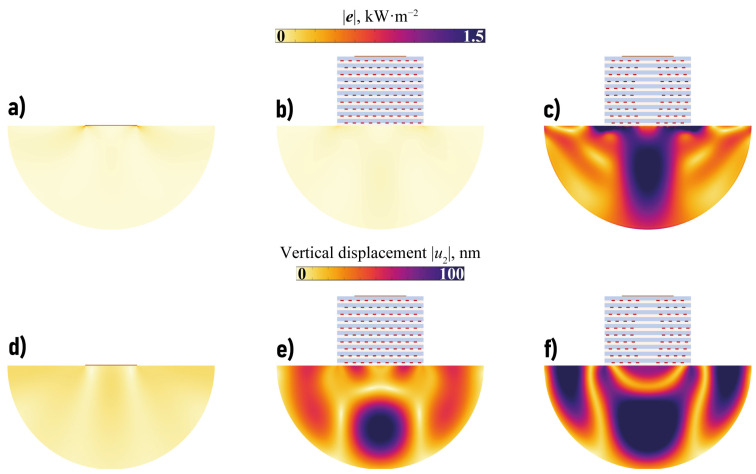
The power density vector |e(x)| and the vertical displacement $|u_{2}\left(x\right)|$ in the substrate in the case of the actuator without EMM intermediate (**a**,**d**), with EMM with voids in hexagonal lattice (**b**,**e**) l=1.75 mm and in oblique lattice with rhombic channel (**c**,**f**) at f=60 kHz.

Figure 10 shows the transferred wave energy $E^{\mathrm{focus}}\left(f\right)$ for the considered configurations with EMMs with voids. Though the plot of $E^{\mathrm{focus}}\left(f\right)$ strongly oscillates, some frequency ranges where the amount of the focused wave energy is sufficiently larger than in the case of a piezoelectric actuator without EMM can be distinguished. For example, such frequency ranges are f∈[60,110] kHz and f∈[1120,1190] kHz for the three configurations with crack-like voids (the second range corresponds to a longitudinal band-gap). Figure 11 and Figure 12 show the power density vector |e(x)|, components of the displacement vector at ω=1138 kHz. Figure 11g–i reveals that periodic array of voids in a hexagonal lattice can strongly increase the amplitudes of vibrations in the substrate even compared to EMM without voids in the longitudinal band-gap.

**Figure 13:**
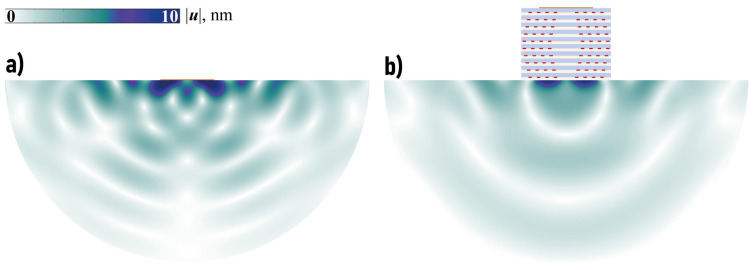
The distribution of amplitudes of the displacement vector u for Hann-windowed signal at $f_{0}=1138$ kHz without EMM at t=0.7 ms (**a**) and with EMM with voids at t=0.95 ms (**b**).

The possibility of wave energy focusing at lower frequencies using EMM with voids, which cannot be provided employing EMM without voids, is demonstrated in Figure 12. It also reveals that wave energy can be accumulated and focused in the substrate at a certain depth. Of course, a transient solution with the input signal spectrum in the vicinity of f=60 kHz must also be examined. The observed focusing effect is demonstrated in Figure 9, but it is not as pronounced as in the previous case, cf. Figure 13. Nevertheless, the introduction of EMM intermediate plays an important role and allows for wave energy guiding into a desirable direction ($x_{2}\to -\infty$).

## 5. Conclusions

In this theoretical study, semi-analytical methods along with the FEM have been applied to investigate the applicability of layered EMM with and without periodically situated crack-like voids for transducer performance enhancement. Since mode conversion between dissimilar wave modes, e.g., longitudinal and transverse waves, is often useful for industrial applications, so several kinds of layered EMMs have been examined as an interlayer converting input elastic energy into transverse waves. The analysis has shown that the proposed configurations with EMM insertion can sufficiently change wave energy transmission from a piezoelectric active element into media for various frequency ranges (relatively low frequencies as well as higher ones). Besides, the bandwidth of the considered transducers with EMM is larger especially at lower frequencies and it can be adjusted via the variation of the parameters of the EMM intermediate.

Typical bulk wave ultrasonic transducers employed in engineering practice are resonance-based and, therefore, of a narrow-band nature. On the other hand, thin piezoelectric films, which are directly attached to the structural surface and could be used for ultrasonic wave excitation due to the high values of their natural frequencies are usually treated as broad-band actuators. As follows from our numerical studies, the transducer with the proposed metamaterial matching layer preserves a rather broad operation range in the frequency domain (e.g., thin lines compared to the thick one in Figure 10—frequency bands between 600 and 900 kHz and between 1100 and 1400 kHz) typical for thin piezoactuators, and, at the same time, for certain frequency bands, the energy flow to the structure is additionally enhanced. The spatial resolution of the transducer could be improved due to the capability of the metamaterial-based matching layer to provide a focused wavefield to the elastic substrate at the prescribed frequency ranges. If frequency resolution is essential, it could be controlled by the proper design of the metamaterial matching layer in the sense of the choice of the materials from which it is composed, and the geometry of periodic defects introduced into its structure.

Since the sensitivity of the reception of elastic waves on the surfaces of some materials by ultrasonic transducers/sensors is relatively small in certain frequency ranges, the proposed configuration or its enhanced version can be considered in the next studies as a sensor with higher sensitivity in certain frequency ranges or for demultiplexing different kinds of elastic waves.

## Figures and Tables

**Table 1:** Material parameters.

Material	Elastic Constants	Piezoelectric Constants	Dielectric Constants	Density [kg/m^3^]
	[GPa]	[C/m^2^]	10^−9^[F/m]	
Aluminium	λ=51.1	—	—	2700
(Material A)	μ=26.3			
Epoxy	λ=0.227	—	—	1200
(Material B)	μ=1.396			
PIC155	$C_{1111}=120$	$e_{211}=-7.24$	$\varepsilon_{11}=9.12$	7800
	$C_{1112}=67.3$	$e_{212}=13.77$	$\varepsilon_{22}=7.55$	
	$C_{2222}=94.4$	$e_{112}=11.91$		
	$C_{1212}=22.3$			

## Data Availability

Data are contained within the article.
